# Beyond financial incentives: a quantitative study on spatial stigma and Puerto Rican physician migration to the United States

**DOI:** 10.1080/17441692.2025.2467767

**Published:** 2025-02-20

**Authors:** Nelson Varas-Díaz, Sheilla R. Madera, Mark Padilla, Torsten B. Neilands, John Vertovec, Kariela Rivera-Bustelo, Adrián J. Santiago-Santiago, Claudia A. Mercado Ríos, Genevieve Reid, Kevin Grove, Alíxida Ramos Pibernus

**Affiliations:** a Global and Sociocultural Studies, School of International and Public Affairs, Florida International University, Miami, FL, USA; b Division of Prevention Science, Department of Medicine, University of California at San Francisco, UCSF, San Francisco, CA, USA; c Behavioral Science Research Institute, Coral Gables, FL, USA; d School of Behavioral and Brain Sciences, Ponce Health Sciences University, Ponce, Puerto Rico

**Keywords:** Physician, migrationg, Puerto Rico, stigma, good health and well-being

## Abstract

Puerto Rico faces a significant health crisis due to the mass migration of physicians to the United States, exacerbating the challenge of achieving the World Health Organisation’s recommended physician-to-population ratio. While economic factors such as higher salaries in the US have been identified as primary drivers, the complexity of this migration wave requires a deeper exploration. This study quantitatively examines the role of push factors, pull factors, and spatial stigmatisation in physician migration from Puerto Rico. Using data from a randomly selected sample of 550 physicians (255 who had migrated to the US and 295 who lived in Puerto Rico), we analyse how perceptions of Puerto Rico’s image and reputation, combined with stigmatisation linked to practicing medicine on the Island, influence migration decisions. Findings highlight that while better economic opportunities in the US are significant, the spatial stigma associated with Puerto Rico’s healthcare system plays a crucial role in the decision to migrate. Policies aimed at curbing physician migration must address not only economic incentives but also the broader socio-cultural perceptions that contribute to the stigmatisation of practicing medicine in Puerto Rico. This study provides insights to inform comprehensive policy solutions to the healthcare crisis in Puerto Rico.

Puerto Rico (PR) is in the midst of an ongoing health crisis marked by the mass migration of physicians to the United States (US). A medical workforce that in 2009 included 14,500 physicians had, by 2020, dwindled to 9,000 (Varney, 2018). This migration pattern, which is aligned in time with the Island’s most recent economic crisis and heightened by the aftermath of Hurricane María in 2017, threatens the possibility of ever reaching the 1/1000 ratio of physicians per capita proposed by the World Health Organisation (Kumar & Pal, 2018). Reports have posited that this migration wave has been led by physicians in specialised areas of medicine, which have become hard to find on the Island (Respaut, 2016; Stephano, n.d.). The effects have been consistently described by local and international news outlets, who have reported that patients frequently have to wait months to receive services (Ávila-Claudio, 2024; Lafarga Previdi et al., 2020; Murphy Marcos, n.d.; Parés Arroyo, 2022; Press, 2016; Rodríguez, 2024; Rosado Lebrón, 2024a; Santiago-Santiago et al., 2024). Research that helps to better understand the underlying forces driving this wave of physician migration is urgently needed to develop effective strategies to curtail it. This urgency is shared by other researchers in diverse settings, as evidenced by a growing amount of published literature that sheds light on physician migration.

## Physician migration as a growing concern

The problem of physician migration seems to be ever-growing and has called the attention of researchers throughout the world and from varied perspectives. The challenges are more concerning for some geographies, as high emigration rates among healthcare professionals are a common challenge in many resource-limited regions. For example, Akl et al. (2007) identified such migratory dynamics in Lebanon, where oversaturation of the job market and limited local training opportunities propel medical graduates to seek better prospects abroad. Hajian et al. (2019) have also identified economic instability and inadequate professional opportunities as important drivers for health worker migration in many developing countries.

More recently, the COVID-19 pandemic has also been identified as an important factor that placed unprecedented burdens on healthcare workers globally and motivated migration patterns among this population. For example, Pszczółkowska et al. (2024) found that systemic failures during the pandemic in Poland led to increased emigration intentions among junior doctors. In Portugal, Ramos and Alves (2017) documented intentions to migrate among resident physicians in training, motivated by dissatisfaction with local work conditions and career prospects. These findings continue to highlight that migration is influenced by a complex interplay of professional, social, and economic factors.

These examples of recent research on physician migration shed light on the complex dynamics that are embedded in the decision to migrate. Individual-level motivators (e.g. desire to advance a career), socio-structural factors (e.g. lack of training sites, low salaries), and cultural pressures (e.g. positive characteristics ascribed to the destination) all play important roles in the decision to leave in search of better opportunities. What these studies also point out is the unequal burden faced by low and middle-income countries throughout the Global South who see their healthcare personnel leave for settings in the Global North, to the detriment of their local communities.

While sharing some of these challenges, PR faces a unique situation linked to its historical context. The problem of healthcare worker migration is facilitated by the fact that US citizenship enables easier movement between the Island and the continental US, allowing professionals to relocate with fewer barriers. This ease of movement significantly contributes to the outflow of healthcare workers from multiple areas of service. For example, Giraldo-Santiago et al. (2024) documented how mental health professionals migrated to the continental US due to dissatisfaction with income, emotional exhaustion, and a perceived lack of organisational support at the local level. These studies help us situate the specific case of physician migration from PR to the US in a wider context, where countries throughout the Global South lose their workforce due to migration to the Global North (Adovor et al., 2021; Moullan, 2014; Saluja et al., 2020).

Based on prior ethnographic research by our team focusing on the PR healthcare system amidst structural collapse post-María (Padilla et al., 2021; Rodríguez-Madera et al., 2021; Varas-Díaz et al., 2022), our team identified a unique phenomenon that frames the migratory decision making of Puerto Rican physicians, which we have referred to as *spatial stigma*. Drawing from a broad theoretical and social science literature that has linked historical disinvestment in disparaged or colonised places to the migration and mobility of its residents (Dummer, 2008; Keene & Padilla, 2010, 2014; Livingston, 2012; Street, 2014), spatial stigma calls for analytic attention to the symbolic underpinnings of physician migration when doctors perceive that their professional status or career aspirations are undermined if they conduct medical training or practice in PR. Our prior qualitative research provides ample supporting evidence for the assertion that spatial stigma is a salient construct in physician migration decisions from PR to the US, leading our team to investigate this more systematically in a randomised physician survey of Puerto Rican physicians, the results of which we report in this article.

## Medical migration and spatial stigma among Puerto Rican physicians

Any investigations into physicians’ decisions to migrate or stay in PR must consider the contextual factors contributing to the crisis. The Caribbean Island archipelago has a Spanish-speaking population of 3.1 million, whose history is starkly marked by colonialism. After more than 400 years of being a Spanish colony, PR was ceded to the United States in 1898 through the Treaty of Paris, which ended the Spanish-American War (Fernández, 1996; Morales, 2019). Since then, it has been an unincorporated territory of the US. Although Puerto Ricans have been US citizens since 1917, the population does not have US citizenship’s full legal and political rights. The Island has faced the effects of US colonisation via the imposition of a foreign language in the education system, medical experimentation with its population, and the use of lands for military training. These manifestations of colonialism are not a thing of the past, but rather practices of the present.

For example, Puerto Rico’s economy, which has been set up as a captive market for US corporations, including health insurance companies, has resulted in a 72-billion-dollar external debt (López-Santana, 2022; Zambrana, 2021). Most of the debt is now negotiated as part of one of US history’s most extensive public debt restructuring efforts. The Island’s economic peril is reflected in the high poverty (39.6%) and unemployment (5.5%) rates (Department of Labor and Human Resources, 2024; US Census Bureau, 2024). The sustained economic crisis, which has intensified since 2006, has significantly affected Puerto Rico’s infrastructure, including its healthcare system (Padilla et al., 2021; Rodríguez-Madera et al., 2021). As mentioned in the latest research reports on the subject, the precarious healthcare system has been described as the root cause for continued negative health outcomes years after Hurricane María (Barba et al., 2023; Benach et al., 2019; Hernández, 2023; Lerman, 2019). Against this backdrop, intensified by the economic strain faced by the people of PR, the local government has relied on economic incentives to curb physician migration, hoping that a lower tax bracket (4%) will deter professionals from leaving or encourage others to return. This policy has been received with mixed opinions, with some physicians expressing criticism and suggesting the problem is more complex, extending beyond economic factors (Montalbám Ríos, 2024a; notiseis360pr, 2024; NotiUno, 2019; Parés Arroyo, n.d.; Varas-Díaz et al., 2023).

Research aimed at better understanding the complex process underlying physician migration from PR to the US has begun to emerge and has effectively used qualitative data-gathering techniques (i.e. in-depth interviews and ethnography) to yield essential data on the topic. For example, qualitative research has examined the factors that motivate physicians to leave PR, commonly known as *push factors*. These include the perception that the quality of life on the Island, in general, has deteriorated, that the healthcare system is rigged in favour of private insurance companies, and that there is a lack of opportunities to complete medical training there (Varas-Díaz et al., 2023). The same research has also begun to document the factors within the US setting that motivate this migration, also known as *pull factors*, and explored how they are frequently linked, among other issues, to better training opportunities. Qualitative research has also served to document the less explored side of the medical migration problem: those who stay. The latter research has added a new layer of complexity: those who do not migrate are often motivated by a sense of attachment to the Island, familial connections, commitment to the people of PR, and a sense of unease about integrating into US culture (Madera et al., 2023).

Finally, qualitative and ethnographic data have uncovered what seems to be a growing stigmatisation of place, or *spatial stigmatisation*, in which physicians feel negatively judged for having their medical training and practice trajectories linked to PR and what is perceived as an inferior medical system (Padilla et al., 2024). Many PR physicians in this prior research defined their career aspirations in reference to a broader, global medical community within which they experienced professional and social stigmatisation for their connections to PR – a colonised island increasingly disparaged for its presumed backwardness, crumbling economy, and ‘unmodern’ medical infrastructure (Padilla et al., 2024). Such dynamics have been echoed in the medical anthropological literature among healthcare personnel in other Global South locations, such as Papua New Guinea (Street, 2014) and Botswana (Livingston, 2012), where physicians must adapt their medical practices to conditions of extreme scarcity while also responding to the gaze and judgment of a global medical system in which they are often perceived as inferior.

Unfortunately, quantitative research on physician migration in PR has lagged behind such qualitative studies and, to the best of our knowledge, is non-existent at the time of this writing. We posit that two factors drive this lack of quantitative inquiry: first, the challenges of systematically recruiting physicians who have emigrated, as they are highly mobile and have limited time to engage in research; and second, the absence of locally developed and validated quantitative measures to assess innovative constructs that may contribute to migration, such as spatial stigma. The quantitative study reported here aimed to address both gaps and incorporates a novel scale of spatial stigma based on prior qualitative research.

These research efforts have set the groundwork for the subsequent exploration of pertinent variables associated with physician migration from a quantitative perspective, which can contribute to the overall understanding of the problem and help develop informed policy solutions, which until now have been limited to exclusively economic approaches undertaken by the Puerto Rican government. Quantitative methods, particularly when attentive to emerging qualitative findings, can provide an additional, more systematic, and statistically representative evidence base to inform policy discussions to address the physician migration crisis. Thus, this study aimed to quantitatively document the role of push factors, pull factors, and spatial stigmatisation in physician migration from PR to the US, aiming to inform future policy initiatives.

## Method

The data presented in this paper stem from an NIH-funded mixed methods study (1R01MD014188) that aimed to document the factors associated with physician migration from PR to the US and its impact on the Island’s healthcare system. The research team used semi-structured qualitative interviews, ethnographic observations, and brief surveys as data collection techniques. This paper focuses on the quantitative data gathered via a brief online survey completed by physicians.

### Participants

A total of 550 physicians participated in the quantitative phase of our study. Participants recruited were between the ages of 25 and 83, and more than half were male (57.1%). Table 1 presents detailed socio-demographic information. The inclusion criteria applied to this sub-sample of the study were (a) having been a licensed physician in PR and/or the US during the past decade, and (b) currently providing medical services in the US or PR. The sample was divided between those who had migrated to the US (*n* = 255) and those who lived in PR (*n* = 295).

### Procedure

Data were collected from June 2021 to August 2022. We began the recruitment process once the team obtained authorisation from the (institution is anonymised for review) IRB (Protocol Approval #108,848). Participants were randomly selected from the Medical Licensing Board of Puerto Rico’s official list of licensed physicians, which at the time included 10,970 individuals. This list is the most comprehensive and regularly upkept registry of physicians licensed to practice medicine in PR and, therefore, the best source to identify the universe of potential participants in our study. The list included some demographic information on physicians, including their current place of practice. It should be noted that physicians who move to the US and retain their license to practice in PR are still included on this governmental list and, at the time of our request, there was no record of a physician cancelling their license. Therefore, our team felt confident that the list of physicians who were licensed to practice medicine in PR but resided in the US was accurate according to local standards.

To reduce selection bias, our team divided the total number of physicians in the database into two subgroups: those living in the US and those in PR, based on their practice locations. Then, the orders of names for both subgroups were randomised using SPSS. To the best of our knowledge, this is the first study on physicians’ migration from PR to the US to use this comprehensive sampling frame and randomisation process.

Our team emailed the physicians in order of appearance in the randomised list. The email included a description of the study, details about their participation process, and a link to a Qualtrics survey. We informed them they were invited to complete a brief survey, which would take approximately 20 min. Afterward, the team contacted physicians by phone at their offices to ensure they had received the invitation email. We called and/or emailed their offices a maximum of three times before moving on to the next potential participant in the randomised list. This strategy allowed us to avoid the potential of participants feeling coerced into engaging with our study and, thus, further reduced potential selection bias. Those who completed the survey received a $50 incentive in the form of an Amazon gift card, a minimal amount in the context of these professionals which allowed us to foster participation without engaging in undue influence. A total of 4,878 invitations were distributed to potential participants, achieving an overall response rate of 11%. Among these, 2,045 invitations were sent to physicians residing in the United States, with 255 completing the survey (12% response rate). Likewise, 2,833 invitations were sent to physicians in Puerto Rico, resulting in 295 responses (10% response rate). These similar rates indicate comparable levels of engagement between the two groups. These response rates align with previous reports indicating low participation rates in research among physicians, a group historically recognised as difficult to engage in such studies (Audibert et al., 2020; Cunningham et al., 2015).

### Measures

Our team developed a brief survey (44-56 items, depending on skip logic) composed of two inventories and a scale to address four areas of interest for our study. This allowed us to gather information on (1) demographic characteristics (nine items), (2) push factors that fostered migration from PR (11 items), (3) pull factors that motivated migration to the US (12 items), and (4) spatial stigma (20 items – see appendix 1) linked to their country of origin. The push factors inventory included questions regarding Puerto Rico’s economic crisis, lack of job opportunities on the Island, scarcity of training opportunities, and exposure to natural disasters. The pull factors inventory included questions on better economic and training opportunities in the United States. (See Table 2) The spatial stigma scale, which was developed specifically for this study, included items addressing negative attitudes towards working and training as a physician in PR, feeling stigmatised for having trained/worked in PR, the notion that Puerto Ricans worked less, Puerto Rico’s image or reputation, and an overall negative outlook on Puerto Rico’s future. Data gathered from our qualitative interviews and ethnographic observations were used to modify and refine the survey items. We also conducted cognitive interviews with ten physicians (*n* = 5 migrant and *n* = 5 non-migrant) to ensure participants understood the questions. This allowed us to simplify the wording of three items and eliminate another two. At the end of this process, we had two versions of the survey with minor wording differences to account for the physician’s location at the time of participation (US or PR). We also had both English and Spanish versions of the survey.

### Data analyses

One-way frequency tables for categorical variables, and measures of central tendency and variability (e.g. median, interquartile range) were computed using SPSS version 24 to characterise the sample.

Part of the survey instrument contained 20 new survey items designed to measure the stigma associated with practicing (or having practiced) medicine in PR and the stigma associated with being a resident or former resident of PR. Because the spatial stigma scale items were new, we performed exploratory factor analysis (EFA) to determine whether one or more latent factors measured by multiple items could be extracted to form subscales for use in later regression analyses. EFA on the 20 spatial stigma items was performed using the freeware program FACTOR 12.04.01 (Ferrando & Lorenzo-Seva, 2017). FACTOR was chosen for this analysis due to its wide variety of features for performing EFA. Prior to extracting factors, a Kaiser-Meyer-Olkin (KMO) test was performed to evaluate the suitability of the correlation matrix of items for EFA. Descriptive analyses revealed evidence of mild kurtosis in the ordinal items’ distributions, so a robust diagonally weighted least squares (DWLS) estimator was chosen to factor a polychoric correlation matrix. Parallel analysis (PA) was used to select the number of retained factors (Auerswald & Moshagen, 2019). Items whose largest factor loadings were greater than or equal to |0.45| were retained (Comrey, 1973). Missing data were addressed by listwise deletion, yielding *n* = 534, a reduction of only 3% from the original *N* of 550.

Following EFA to extract latent factors, we fitted confirmatory factor analysis (CFA) models to evaluate the fit of the EFA-based solution when cross-loadings were fixed to zero and to refine the EFA-based subscales (Gerbing & Hamilton, 1996). CFA models were fitted using the WLSMV estimator, a DWLS method, in M*plus* version 8.10 (Muthén & Muthén, 2023). The exact fit of CFA models to the data was tested using the chi-square test of exact data-model fit. Because the chi-square test of exact fit tends to detect trivial departures from perfect model-data fit in most real-world applications of CFA (Bollen & Long, 1993), the following well-studied descriptive indices of approximate model fit were used to evaluate model-data fit: the standardised root mean square error (SRMR), Bentler’s comparative fit index (CFI), and the root mean square error of approximation (RMSEA). Specifically, CFA model fit was determined by Hu and Bentler’s recommended multi-index criteria utilising a combination of these statistics: SRMR ≤ 0.08 and either (a) RMSEA ≤ 0.06 *or* (b) CFI ≥ 0.95 signified a satisfactory approximate CFA model-data fit (Hu & Bentler, 1999). Items with low factor loadings that were also deemed unnecessary to retain on a conceptual basis by the study’s principal investigators were dropped at this stage, yielding the final subscales. Cronbach’s alphas were then computed for each subscale using Stata version 18 (StataCorp, 2023).

Multiple logistic regression analysis was then performed using M*plus*. The outcome variable was migration to the US (0 = did not migrate; 1 = migrated). Explanatory variables included push factors, pull factors, participant age (in years), gender (1 = male; 2 = female), generalist vs. specialist professional designation (1 = generalist; 2 = specialist), and income in dollars per year divided by $100,000 so that the rescaled income variable was on a similar scale to the other explanatory variables to facilitate model convergence. To highlight the value added by the novel spatial stigma subscales, we fitted a second multivariable logistic regression model containing all the previously listed explanatory variables plus the spatial stigma subscales.^1^

In addition, a single transgender-identified participant was excluded because the inclusion of a third gender category containing a single observation created model instability. Incomplete data was generally modest, with 92% of cases (*n* = 504) having complete data and all variables except for income (7% missing) having less than 1% percent missing. However, the aggregate loss of cases with partial information would result in a reduction of the analysis *n* from 549 to 504, a reduction of 8%. The recommended thresholds for loss of cases below which missing data can be ignored and addressed using listwise deletion typically range from 5% to 10%. To maximise rigor, utilise all available information from each participant, and include participants with partial data as well as those with complete data in the analysis to improve the generalizability of inferences, we used direct maximum likelihood estimation for the logistic regression analyses (*n* = 549) (Enders, 2022). For each explanatory variable, we report the adjusted odds ratio (aOR), its 95% confidence interval (95% CI), and the *p*-value associated with the test of the null hypothesis that the odds ratio equals 1.00 in the population. Alpha was set at 5% for hypothesis testing.

### Statistical power analysis

We used NCSS PASS to compute the minimum detectable effect size based on a planned sample size of 550 physicians. The objective of the power analysis was to determine whether our planned sample size would be sufficient to estimate small to medium effects for our planned multivariable logistic regression analysis, as defined using Cohen’s benchmarks of the standardised effect size value *h* being between *h* = 0.20 (small effect size) and *h* = 0.50 (medium effect size). Assuming N = 549, alpha = 0.05, power = 0.80, 46% of participants having migrated to the US, and a multiple *R* of 0.30 among covariates, the minimum detectable *h*-value of a continuous stigma scale or push/pull score was 0.13, a small standardised minimum detectable effect size.

## Results

### Exploratory factor analysis (EFA) results

The Kaiser-Meyer-Olkin (KMO) test value was 0.75, which indicated sufficient factorability of the correlation matrix. Parallel analysis extracted three factors. The first factor contained items measuring *work context* (e.g. a participant was stigmatised due to their training or employment history in PR). Two items were dropped due to low factor loadings: ‘How frequently do you think that it would be difficult to obtain new career opportunities for physicians who have worked in Puerto Rico?’ (loading = 0.31) and ‘How frequently do you think that it would be difficult to obtain new career opportunities for physicians who studied in Puerto Rico?’ (loading = 0.39). The second factor, *work productivity*, contained two items measuring respondents’ perceptions of Puerto Ricans as hard-working and productive citizens. The third factor assessed participants’ perceptions of *Puerto Rico’s image* in terms of likely future career opportunities, the quality of the Island’s healthcare system, and the participant’s belief in the Island’s leaders’ and citizens’ ability to overcome crises and build a better country in the future (see Table 3 for a list of all 20 stigma items). Three items were dropped from this subscale: ‘How much has Puerto Rico’s image or reputation been damaged by recent natural crises (e.g. earthquakes, hurricanes) the Island is facing?’ (loading = 0.40), ‘How much do you think Puerto Rico is a beautiful place to live?’ (loading = 0.37), and ‘How much would a physician’s career benefit from studying medicine in Puerto Rico?’ (loading = 0.23).

A test of the null hypothesis of the exact fit of the initial CFA based on the EFA’s factor structure was rejected (*χ*^2^(87) = 805.10, *p* < 0.0001). However, the CFA fit the data satisfactorily on an approximate basis based on Hu and Bentler’s criterion of SRMR < 0.08 and CFI ≥ 0.95 (SRMR = 0.074; CFI = 0.959; RMSEA = 0.123). Examination of the standardised factor loadings revealed all were moderate to large (i.e. > |0.58|), except for one item, ‘How much has Puerto Rico’s image or reputation been damaged by recent economic crises the Island is facing?’ whose factor loading was 0.33. Accordingly, based on its low factor loading and its focus on the economy rather than the specific context for medical workers, we removed the item and refitted the CFA. A test of the null hypothesis of the exact fit of this revised CFA was rejected χ^2^(74) = 749.55, *p* < 0.0001. However, the revised CFA fit the data satisfactorily on an approximate basis based on Hu and Bentler’s criterion of SRMR ≤ 0.08 and CFI ≤ 0.95 (SRMR = 0.074; CFI = 0.961; RMSEA = 0.129) with all factor loadings greater than or equal to 0.58 (see Table 3). Reliability analysis of each subscale revealed satisfactory alphas for work context (*α* = 0.79), work productivity (*α* = 0.93), and Puerto Rico’s image (*α* = 0.81).

### Logistic regression analysis results

Multivariable logistic regression results appear in Table 4. Higher numbers of reported push factors were associated with lower odds of having migrated whereas the odds of having migrated were higher for higher numbers of pull factors. Age was not statistically significantly associated with the odds of having migrated, but female-identified participants had lower odds of having migrated, though only when spatial stigma was taken into account. Specialists had lower odds of having migrated than generalists while higher income was associated with higher odds of having migrated.

Spatial stigma contributed statistically significant effects above and beyond the push factors, pull factors, and participant characteristics listed in the previous paragraph. Specifically, higher work context stigma and work productivity scores were associated with having lower odds of having migrated. However, higher Puerto Rico image stigma scores were associated with higher odds of having migrated.

## Discussion

The results from our quantitative exploration of the role of push factors, pull factors, and spatial stigma in physician migration in PR yield important initial insights that can help researchers, practicing health professionals, and policymakers on the Island and the continental US better understand the complexity of physicians’ migration decisions. For example, push factors within the Island, local spatial stigma related to work context, and perceptions of a lack of productivity in the setting were associated with being a resident of PR versus the US. These variables are frequently discussed in the press as motivators to migrate, but in our sample, they were associated with staying in PR. It seems that for many, these may not be enough to foster migration or that a larger cultural and emotional pull could be functioning as a buffering element motivating physicians to stay in place despite their awareness of situations and experiences that could, in other scenarios, encourage them to migrate. These quantitative findings echo the results from our qualitative studies on this matter, which have found that for some physicians sense of place attachment, which is influenced by a love of local culture, emotional bonds with place and family, and concerns over integrating into US culture, play a crucial role in deciding to stay in PR despite the hardships endured (Madera et al., 2023). We would also argue that those who remain bear the brunt of the negative experiences faced by physicians and are more exposed to local stigmatising attitudes towards place; thus, these variables become salient for them. This finding is counterintuitive to the local discussions taking place in PR over media channels and in policy circles that aim to explain physician migration in PR and, thus, merit detailed attention moving forward.

Increased odds of practicing in the US were associated with US-based pull factors, Puerto Rico’s negative image, and higher income. Puerto Rico’s negative image – a component of our spatial stigma measure – stands out as an important finding because it is linked to an overall assessment of the Island and its lack of potential to overcome the ongoing crisis. These quantitative findings are in line with our qualitative research on this topic, which has highlighted how an overall sense of deterioration of the country and the perception that the healthcare system is rigged or corrupt are important factors motivating physicians to leave (Santiago-Santiago et al., 2024; Varas-Díaz et al., 2023).

More importantly, the questions in our stigma scale linked to Puerto Rico’s negative image shed light on important concerns physicians can have over the Island’s potential to position itself as a good place to practice medicine. If the sense is that PR is not a good place to train and practice medicine, then the potential for stigma to drive more migration remains an ongoing concern in addressing the crisis of physician loss in PR. As long as physicians feel that they cannot practice in the Puerto Rican setting to the best of their abilities due to contextual constraints and negative perceptions by the global medical community, then individual-level focused policy solutions (e.g. economic incentives) may not be enough to curtail the migratory wave.

While our cross-sectional methodological approach and analysis process cannot be used to establish causal relations, the finding is important as efforts to curtail physician migration and incentivize those who have left to return to the Island will need to address not only issues of salary equity but also the reduction of the spatial stigma attached to PR, which is informed by perceptions of a bleak future, infrastructural challenges, and the inability to develop a successful medical career in this setting. It bears repeating that this quantitative result is also consistent with existing qualitative evidence suggesting place-based stigma is an unexamined factor in the migration crisis. Furthermore, our findings seem to contradict the prevailing local narrative put forth by professional organisations and the media, which emphasises that physician migration is primarily composed of specialists rather than generalists. This finding merits further attention and longitudinal follow-up, as it may indicate potential shifts in the types of physicians leaving PR. It may be that generalists are finding easier pathways to practice medicine in the continental US due to ease of recruitment, that the discontent with the local context has driven both specialists and generalists to search for better opportunities, or that these physicians have become valued in the continental US due to reasons beyond their specialties (e.g. bilingual abilities).

We are aware that such future studies with physicians face multiple challenges, including the fact that this population is hard to reach, has little time to engage in studies, and that the migration process continues to escalate as we write these results. We believe that highlighting some of the strengths and limitations of our approach could help other researchers efficaciously navigate future endeavors. Thus, we understand that the reported study has several notable strengths. The randomisation process allowed us to reduce bias in the selection of the sample of both migrant and non-migrant physicians living in both the origin and destination sites, enhancing the validity of the findings. Additionally, the study’s development and initial validation of a novel spatial stigma scale provided unique insights into the socio-cultural factors influencing migration, which are often overlooked in similar studies. This newly developed scale is expected to enable future researchers to assess an emerging construct in the Puerto Rican context, which evidence suggests is a significant factor for understanding physician migration. Future efforts would benefit from replicating methods that help us focus on the less explored individual and cultural dimensions that foster their migration. For example, in the Giraldo-Santiago et al. (2024) study with mental health professionals in PR, age was a significant variable associated with migration thoughts. In our sample, age was not significant, and such differences suggest that future studies should look at age as a variable and how it impacts migration.

Our study also had limitations that should be surpassed in future efforts. Our cross-sectional design limits the ability to infer causal relationships between identified factors and migration decisions. Also, despite efforts to minimise selection bias, there remains the possibility that non-respondents may differ in significant ways from participants, potentially affecting the representativeness of the sample. Lastly, the study’s reliance on self-reported data introduces the risk of recall bias, which could influence the accuracy of the reported push and pull factors. Future research should take into consideration these limitations by incorporating longitudinal designs and triangulating self-reported data with objective measures.

In summary, as the government of PR continues to develop and implement strategies to reduce medical migration, it would do well to understand that the solutions to the problem are not solely economic. This fact has been described by local physicians, as reported in previous research (Giraldo-Santiago et al., 2024; Santiago-Santiago et al., 2024). To date, the only policy initiative implemented to curtail the migration wave has been to offer tax credits to practicing physicians, a strategy that has not benefited the whole medical class due to limitations imposed by the US Fiscal Control Board, which manages the local budget (Hernández Cabiya, 2023; Irizarry Álvarez, 2019; Montalbám Ríos, 2024b; Rosado Lebrón, 2024a, 2024b). Still, the findings from our study shed light on a more complex scenario in which better remuneration is but one of the motivating factors behind the migration process. Thus, policymakers must develop more sophisticated muti-level strategies to address physician migration in PR. Based on our previous research, and the findings reported in this article, these strategies should include, among others, the reduction of the overarching control insurance companies hold over the health care system and its practitioners, creation of more positions for training residents who decide to stay in PR upon graduation, and the development of policies to address spatial stigma and its linkages to healthcare provision on the Island (e.g. media campaigns, linkages with physicians in the diaspora to facilitate their return) (Santiago-Santiago et al., 2024). Spatial stigma may also be addressed by highlighting the local strengths of PR’s broader medical system in the face of scarcity and environmental catastrophe, which includes innovative mutual aid and community-level initiatives that should be lauded and championed.

To do so, policymakers, healthcare facility administrators, physicians’ professional organisations, patient organisations, private insurance companies, and social scientists, just to name a few of the actors with a stake in this problem, will do well to contextualise the physician migration problem from PR to the US in a larger global context. By this, we mean the systematic depletion of human resources taking shape throughout the Global South by the Global North, which becomes a new form of extractivism that needs to be reflected and acted upon (Farthing & Fabricant, 2018). If left unattended, this practice, now carried out with highly specialised human resources, will continue to echo vestiges of colonial dynamics that have plagued the field of medicine and health (Bashford, 2003).

## Supplementary Material

Supp 1

## Figures and Tables

**Table 1.** Survey respondents’ demographic information (*N* = 550)^a^

Variable	US-based Respondents		PR-based Respondents	
	*n*	%	*n*	%
Where do you mainly practice medicine?				
US	255	100.0	n/a	n/a
PR	n/a	n/a	295	100.0
Most common living locations (States (US) and Municipalities (PR))				
Florida	149	58.4	n/a	n/a
Texas	17	6.7	n/a	n/a
Massachusetts	7	2.7	n/a	n/a
Arizona	6	2.4	n/a	n/a
Louisiana & New Mexico	5	2.0	n/a	n/a
Other US States	67	26.3	n/a	n/a
San Juan	n/a	n/a	82	27.8
Guaynabo	n/a	n/a	29	9.8
Ponce	n/a	n/a	21	7.1
Caguas	n/a	n/a	17	5.8
Carolina & Mayaguez	n/a	n/a	14	4.7
Other PR Municipalities	n/a	n/a	120	40.7
Preferred Language				
Spanish	168	65.9	234	79.3
English	87	34.1	61	20.7
Age range				
25–40	50	19.6	97	32.9
41–50	80	31.4	72	24.4
51–60	67	26.3	48	16.3
61+	56	22.0	73	24.7
Gender				
Male	159	62.4	155	52.5
Female	95	37.3	140	47.5
Transgender	1	0.4	–	–
Other	–	–	–	–
Sexual Orientation				
Heterosexual	237	92.9	284	96.3
Homosexual/Lesbian	16	6.3	8	2.7
Bisexual	1	0.4	1	0.3
Other	1	0.4	1	0.3
Marital Status				
Single	29	11.4	56	19.0
Married	188	73.7	186	63.1
Divorced	16	6.3	25	8.5
Separated	1	0.4	1	0.3
Widowed	3	1.2	6	2.0
Living with partner (not legally married)	17	6.7	19	6.4
Other	1	0.4	2	0.7
Annual Income				
≤$100,000	22	8.6	95	32.2
$101,000-$200,000	67	26.3	92	31.2
$201,000-$300,000	100	39.2	42	14.2
$301,000-$400,000	24	9.4	19	6.4
$401,000+	24	9.4	26	8.8
Generalist or Specialist^b^				
Generalist	174	68.2	156	52.9
Specialist	81	31.8	138	46.8
Years of Experience (in the US or PR, respectively)				
≤5 years	83	32.5	78	26.4
6–10 years	72	28.2	32	10.8
11–20 years	58	22.7	68	23.1
21 + years	40	15.7	114	38.6
Did you practice medicine in PR at any time in your career (including fellowships or internships)?				
Yes	230	90.2	n/a	n/a
Did you ever practice medicine in the mainland US?				
Yes	n/a	n/a	77	26.1

a Demographic responses were optional, except for the first question regarding practice location (i.e. ‘Where do you mainly practice medicine?’). As a result, percentages and *n* values do not always equal 100% of the total *N* for each group (US-based versus PR-based respondents).

b For the purpose of analysis, ‘generalist’ included areas of practice like general or family medicine, and ‘specialist’ included areas of practice like anesthesiology, immunology, and neurosurgery.

**Table 2.** Items in the push and pull factor inventories.

Push Factors that Fostered Migration from Puerto Rico Fewer career opportunities Fewer training opportunities Economic instability Natural disasters (e.g. hurricane Maria, earthquakes) PR’s political status Difficulties with health care infrastructure Reduction of number of patients on the Island Challenges with insurance companies in PR Less institutional support to practice medicine in PR Burnout due to the high number of patients treated in PR Fewer family members in PR
Pull Factors that Fostered Migration to the US Different training opportunities More training opportunities More competitive salaries More benefits (e.g. malpractice insurance, retirement funds) Financial stability More political stability Appropriate healthcare infrastructure Manageable number of patients to treat Easier interactions with insurance companies More institutional support to practice medicine To be closer to other family members In order to pay-off my student loans

**Table 3.** Items and factor loadings for Puerto Rico Spatial Stigma Scale.

Factor/Item	EFA	CFA
Work Context		
1. How frequently do you think that it would be difficult to obtain new career opportunities for physicians who have worked in Puerto Rico?	0.314	–
2. How frequently do you think that it would be difficult to obtain new career opportunities for physicians who studied in Puerto Rico?	0.393	–
3. How frequently do you think that physicians could feel stigmatised because they were employed in Puerto Rico?	0.703	0.800
4. How frequently do you think that physicians could feel stigmatised because they studied medicine in Puerto Rico?	0.788	0.875
5. How much do you think people in the mainland US believe medical services in Puerto Rico are substandard?	0.546	0.641
6. How much do you think physicians in the mainland US believe Puerto Ricans are less prepared to practice medicine?	0.674	0.689
7. How much do you think that studying medicine in Puerto Rico can disadvantage a physician’s career?	0.639	0.749
Work Productivity		
8. How much do you think Puerto Ricans are hardworking citizens?^a^	0.996	0.923
9. How much do you think Puerto Ricans are productive citizens?^a^	0.979	1.010
Puerto Rico’s Image		
10. How much has Puerto Rico’s image or reputation been damaged by recent economic crises the Island is facing?	0.557	–
11. How much has Puerto Rico’s image or reputation been damaged by recent natural crises (e.g. earthquakes, hurricanes) the Island is facing?	0.397	–
12. How much do you think Puerto Rico is a beautiful place to live?^a^	0.365	–
13. How much do you think Puerto Rico can offer you a promising future?^a^	0.772	0.709
14. How much do you think Puerto Ricans have the capacity to overcome the Island’s current economic crisis?^a^	0.515	0.672
15. How probable is it that Puerto Rico’s leaders will build a better country in the future?^a^	0.515	0.576
16. How much do you think Puerto Rico is a good place to develop a medical career?^a^	0.865	0.836
17. How much would a physician’s career benefit from working in Puerto Rico?^a^	0.791	0.772
18. How much would a physician’s career benefit from studying medicine in Puerto Rico?	0.233	–
19. How much would you say that the health care system in Puerto Rico is of high quality?^a^	0.470	0.662
20. How much would you say that the health care infrastructure in Puerto Rico is advanced?^a^	0.471	0.654

Notes: EFA: Exploratory factor analysis performed in FACTOR 12.04.01 (*N* = 534); CFA: Confirmatory factor analysis performed in M*plus* 8.10 (*N* = 550).

a Reverse-scored items.

**Table 4.** Multivariable logistic regression analyses explaining physician migration.

Explanatory Variable	Model without Spatial Stigma Measures *R*^2^ = .233			Model with Spatial Stigma Measures *R*^2^ = .359		
	aOR	95% CI	*p*	aOR	95% CI	*p*
Push Factors	0.795	0.706, 0.896	<.001	0.728	0.636, 0.833	<.001
Pull Factors	1.160	1.053, 1.279	0.003	1.127	1.015, 1.251	0.025
Work Context	–	–	–	0.646	0.493, 0.847	0.002
Work Productivity	–	–	–	0.486	0.385, 0.613	<.001
PR’s Image	–	–	–	3.309	2.143, 4.309	<.001
Age	1.008	0.992, 1.023	0.322	0.998	0.981, 1.104	0.774
Female	0.727	0.498, 1.061	0.098	0.658	0.440, 0.984	0.042
Specialist	0.382	0.255, 0.572	<.001	0.406	0.264, 0.624	<.001
Income	1.358	1.194, 1.545	<.001	1.362	1.193, 1.554	<.001

Notes: The outcome variable is whether the physician migrated (1) vs. did not migrate (0) from Puerto Rico. *N* = 549 for both models. aOR is the adjusted odds ratio. *R^2^* values were calculated using McKelvey and Zavoina’s method (McKelvey & Zavoina, 1975).

## Data Availability

Individual participant data collected will not be available, following ethical guidelines.

## Footnotes

1. Our original analysis featured one multivariable logistic regression model containing all explanatory variables, including the spatial stigma variables. We are grateful to an anonymous reviewer for suggesting the two-model strategy presented here to highlight contributions of the spatial stigma measure above and beyond the other explanatory variables.
