# Supplementary material for: Beyond financial incentives: a quantitative study on spatial stigma and Puerto Rican physician migration to the United States
Source: Glob Public Health. Author manuscript; Available in PMC 2025 Dec 1. (PMC11921220; doi:10.1080/17441692.2025.2467767)
Supplement: Supp 1 [file NIHMS2060251-supplement-Supp_1.docx]

**Appendix 1: Stigma Scale**

**English Version, in order of appearance**

| **Item #** | **Original Question** | **Response Options (with associated score)** | | | | |
| --- | --- | --- | --- | --- | --- | --- |
| 1 | How much has PR's image or reputation been damaged by recent economic crises the Island is facing? | None at all (1) | A little (2) | A moderate amount (3) | A lot (4) | A great deal (5) |
| 2 | How much has PR's image or reputation been damaged by recent natural crises (e.g., earthquakes, hurricanes) the Island is facing? | None at all (1) | A little (2) | A moderate amount (3) | A lot (4) | A great deal (5) |
| 3 | *How much do you think PR is a beautiful place to live? | Not at all (1) | Slightly (2) | Moderately (3) | Very (4) | Extremely (5) |
| 4 | *How much do you think PR can offer you a promising future? | Not at all (1) | Slightly (2) | Moderately (3) | Very (4) | Extremely (5) |
| 5 | *How much do you think Puerto Ricans have the capacity to overcome the Island's current economic crisis? | Not at all (1) | Slightly (2) | Moderately (3) | Very (4) | Extremely (5) |
| 6 | *How much do you think Puerto Ricans are hardworking citizens? | Not at all (1) | Slightly (2) | Moderately (3) | Very (4) | Extremely (5) |
| 7 | *How much do you think Puerto Ricans are productive citizens? | Not at all (1) | Slightly (2) | Moderately (3) | Very (4) | Extremely (5) |
| 8 | *How probable is it that PR's leaders will build a better country in the future? | Definitely won't (1) | Probably won't (2) | Might or might not (3) | Probably will (4) | Definitely will (5) |
| 9 | *How much do you think PR is a good place to develop a medical career? | Not at all (1) | Slightly (2) | Moderately (3) | Very (4) | Extremely (5) |
| 10 | *How much would a physician’s career benefit from working in PR? | Not at all (1) | Slightly (2) | Moderately (3) | Very (4) | Extremely (5) |
| 11 | *How much would a physician’s career benefit from studying medicine in PR? | Not at all (1) | Slightly (2) | Moderately (3) | Very (4) | Extremely (5) |
| 12 | How frequently do you think that it would be difficult to obtain new career opportunities for physicians who have worked in PR? | Never (1) | Sometimes (2) | About half the time (3) | Most of the time (4) | Always (5) |
| 13 | How frequently do you think that it would be difficult to obtain new career opportunities for physicians who studied in PR? | Never (1) | Sometimes (2) | About half the time (3) | Most of the time (4) | Always (5) |
| 14 | How frequently do you think that physicians could feel stigmatized because they were employed in PR? | Never (1) | Sometimes (2) | About half the time (3) | Most of the time (4) | Always (5) |
| 15 | How frequently do you think that physicians could feel stigmatized because they studied medicine in PR? | Never (1) | Sometimes (2) | About half the time (3) | Most of the time (4) | Always (5) |
| 16 | How much do you think people in the mainland US believe medical services in PR are substandard? | Not at all (1) | Slightly (2) | Moderately (3) | Very (4) | Extremely (5) |
| 17 | How much do you think physicians in the mainland US believe Puerto Ricans are less prepared to practice medicine? | Not at all (1) | Slightly (2) | Moderately (3) | Very (4) | Extremely (5) |
| 18 | *How much would you say that the health care system in PR is of high quality? | Not at all (1) | Slightly (2) | Moderately (3) | Very (4) | Extremely (5) |
| 19 | *How much would you say that the healthcare infrastructure in PR is advanced? | Not at all (1) | Slightly (2) | Moderately (3) | Very (4) | Extremely (5) |
| 20 | How much do you think that studying medicine in PR can disadvantage a physician's career? | Not at all (1) | Slightly (2) | Moderately (3) | Very (4) | Extremely (5) |
| *Response options were listed on the survey from least manifested (e.g., “Not at all”) to most manifested (e.g., “Extremely”). However, Stigma Scale items 3-11 and 18-19 were reverse scored for our analysis to reflect stigma levels accurately. These questions asked respondents to describe how positive they viewed Puerto Rico and its health system (e.g., item 18: "How much would you say that the health care system in PR is of high quality?"). A negative response to these questions (e.g., “Not at all” for the previous example) indicated higher levels of stigma. In contrast, positive perceptions of stigma-related beliefs (e.g., "Always" to "How frequently do you think it would be difficult to obtain new career opportunities for physicians who studied in PR?") also scored higher and did not require reverse coding. The reverse-coded items are marked with an asterisk (*) in the table above.  Authorization for Use of the Stigma Scale - Permission is granted to use this Stigma Scale in academic and research settings. When using the scale, users are required to cite this article. For inquiries and additional permissions, please contact the corresponding author. Modifications to the Stigma Scale are allowable; if the Stigma Scale is modified, we request that authors indicate that modifications were performed and what modifications were made to the original scale. | | | | | | |

**Spanish Version, in order of appearance**

| **Item #** | **Original Question** | **Response Options (with associated score)** | | | | |
| --- | --- | --- | --- | --- | --- | --- |
| 1 | *¿Cuánto daño ha sufrido la imagen o reputación de P.R. por las recientes crisis económicas a las que se enfrenta la Isla?* | *Ninguno (1)* | *Un poco (2)* | *Una cantidad moderada (3)* | *Mucho (4)* | *Un gran cantidad (5)* |
| 2 | *¿Cuánto daño ha sufrido la imagen o reputación de P.R. por las recientes crisis naturales (p. ej., terremotos, huracanes) a las que se enfrenta la Isla?* | *Ninguno (1)* | *Un poco (2)* | *Una cantidad moderada (3)* | *Mucho (4)* | *Un gran cantidad (5)* |
| 3 | **¿Cuánto piensa que P.R. es un hermoso lugar para vivir?* | *Nada (1)* | *Levemente (2)* | *Moderado (3)* | *Mucho (4)* | *Extremadamente (5)* |
| 4 | **¿Cuánto piensa que P.R. le puede ofrecer un futuro prometedor?* | *Nada (1)* | *Levemente (2)* | *Moderado (3)* | *Mucho (4)* | *Extremadamente (5)* |
| 5 | **¿Cuánto piensa que los puertorriqueños tienen la capacidad para superar la actual crisis económica de la Isla?* | *Nada (1)* | *Levemente (2)* | *Moderado (3)* | *Mucho (4)* | *Extremadamente (5)* |
| 6 | **¿Cuánto piensa que los puertorriqueños son ciudadanos trabajadores?* | *Nada (1)* | *Levemente (2)* | *Moderado (3)* | *Mucho (4)* | *Extremadamente (5)* |
| 7 | **¿Cuánto piensa que los puertorriqueños son ciudadanos productivos?* | *Nada (1)* | *Levemente (2)* | *Moderado (3)* | *Mucho (4)* | *Extremadamente (5)* |
| 8 | **¿Qué tan probable es que los líderes de P.R. construyan un mejor país en el futuro?* | *Definitivamente no lo harán (1)* | *Probablemente no lo harán (2)* | *Podrían o no (3)* | *Probablemente lo harán (4)* | *Definitivamente lo harán (5)* |
| 9 | **¿Cuánto piensa que P.R. es un buen lugar para desarrollar una carrera médica?* | *Nada (1)* | *Levemente (2)* | *Moderado (3)* | *Mucho (4)* | *Extremadamente (5)* |
| 10 | **¿Cuánto se beneficiaría la carrera de un médico por trabajar en P.R.?* | *Nada (1)* | *Levemente (2)* | *Moderado (3)* | *Mucho (4)* | *Extremadamente (5)* |
| 11 | **¿Cuánto se beneficiaría la carrera de un médico por estudiar medicina en P.R.?* | *Nada (1)* | *Levemente (2)* | *Moderado (3)* | *Mucho (4)* | *Extremadamente (5)* |
| 12 | *¿Con qué frecuencia piensa que sería difícil obtener nuevas oportunidades profesionales para los médicos que han trabajado en P.R.?* | *Nunca (1)* | *Algunas veces (2)* | *Aproximadamente la mitad de las veces (3)* | *La mayoría de las veces (4)* | *Siempre (5)* |
| 13 | *¿Con qué frecuencia piensa que sería difícil obtener nuevas oportunidades profesionales para los médicos que han estudiado en P.R.?* | *Nunca (1)* | *Algunas veces (2)* | *Aproximadamente la mitad de las veces (3)* | *La mayoría de las veces (4)* | *Siempre (5)* |
| 14 | *¿Con qué frecuencia piensa que los médicos pudiesen sentirse estigmatizados porque estuvieron empleados en P.R.?* | *Nunca (1)* | *Algunas veces (2)* | *Aproximadamente la mitad de las veces (3)* | *La mayoría de las veces (4)* | *Siempre (5)* |
| 15 | *¿Con qué frecuencia piensa que los médicos pudiesen sentirse estigmatizados porque estudiaron medicina en P.R.?* | *Nunca (1)* | *Algunas veces (2)* | *Aproximadamente la mitad de las veces (3)* | *La mayoría de las veces (4)* | *Siempre (5)* |
| 16 | *¿Cuánto piensa que la gente en EE. UU. cree que los servicios médicos en P.R. son por debajo de los estándares?* | *Nada (1)* | *Levemente (2)* | *Moderado (3)* | *Mucho (4)* | *Extremadamente (5)* |
| 17 | *¿Cuánto piensa que los médicos en EE. UU. cree que los puertorriqueños están menos preparados para practicar la medicina?* | *Nada (1)* | *Levemente (2)* | *Moderado (3)* | *Mucho (4)* | *Extremadamente (5)* |
| 18 | **¿Cuánto diría usted que el sistema de cuidado de salud de P.R. es de alta calidad?* | *Nada (1)* | *Levemente (2)* | *Moderado (3)* | *Mucho (4)* | *Extremadamente (5)* |
| 19 | **¿Cuánto diría usted que la infraestructura de cuidado de salud de P.R. es avanzada?* | *Nada (1)* | *Levemente (2)* | *Moderado (3)* | *Mucho (4)* | *Extremadamente (5)* |
| 20 | *¿Cuánto piensa que estudiar medicina en P.R. puede poner en desventaja la carrera de un médico?* | *Nada (1)* | *Levemente (2)* | *Moderado (3)* | *Mucho (4)* | *Extremadamente (5)* |
| * Las opciones de respuesta en la encuesta se enumeraron de la menos manifestada (por ejemplo, “Nunca”) a la más manifestada (por ejemplo, “Extremadamente”). Sin embargo, los ítems 3-11 y 18-19 de la Escala de Estigma fueron invertidos en la puntuación para nuestro análisis con el fin de reflejar con precisión los niveles de estigma. Estas preguntas pedían a los encuestados que describieran cuán positivamente percibían a Puerto Rico y su sistema de salud (por ejemplo, el ítem 18: “¿Cuánto diría usted que el sistema de cuidado de salud de P.R. es de alta calidad?"). Una respuesta negativa a estas preguntas (por ejemplo, “Nada” en el ejemplo anterior) indicaba niveles más altos de estigma. Por el contrario, las percepciones positivas de creencias relacionadas con el estigma (por ejemplo, "Siempre" para la pregunta “¿Con qué frecuencia piensa que sería difícil obtener nuevas oportunidades profesionales para los médicos que han estudiado en P.R.?") también recibieron puntuaciones más altas y no requirieron inversión en la codificación. Los ítems con puntuación invertida están marcados con un asterisco (*) en la tabla anterior.  Autorización para el uso de la Escala de Estigma - Se permite el uso de esta Escala de Estigma en entornos académicos y de investigación. Al utilizar la escala, se requiere que los usuarios citen este artículo. Para consultas y permisos adicionales, por favor contacte al autor correspondiente. Se permiten modificaciones a la Escala de Estigma; si la Escala de Estigma es modificada, solicitamos que los autores/as indiquen que se realizaron modificaciones y cuáles fueron los cambios hechos a la escala original | | | | | | |
